# The impact and optimal indication of non-curative gastric resection for stage IV advanced gastric cancer diagnosed during surgery: 10 years of experience at a single institute

**DOI:** 10.1186/s12957-016-0790-z

**Published:** 2016-03-10

**Authors:** Naoya Yamada, Atsushi Akai, Yukihiro Nomura, Nobutaka Tanaka

**Affiliations:** Department of Surgery, Asahi General Hospital, 1326 I, Asahi-shi, Chiba 289-2511 Japan

**Keywords:** Stage IV gastric cancer, Non-curative gastric resection, Postoperative chemotherapy

## Abstract

**Background:**

The survival benefit of non-curative gastric resection for patients with stage IV gastric cancer is still unclear.

**Methods:**

Of the patients who underwent open abdominal surgery that was preoperatively intended to be a radical excision procedure for gastric cancer, 72 were diagnosed with stage IV during the operation. At this institution, non-curative gastric resection is performed whenever possible.

**Results:**

Non-curative gastric resection was performed in 44 of the 72 patients. According to the survival analysis, the median survival times in the gastric resection and no-resection groups were 1.9 and 0.9 years, respectively (log-rank test, *p* = 0.014). Based on the multivariate analysis, we selected gastric resection (hazard ratio [HR] = 0.309; 95 % confidence interval [CI] = 0.152–0.615) and postoperative chemotherapy (HR = 0.136; 95 % CI = 0.056–0.353) as independent factors associated with overall survival (OS). In the subgroup analyses of OS, the factors that were associated with gastric resection having no survival benefit were the existence of distant lymph node or liver metastasis (*p* = 0.527) and the lack of postoperative chemotherapy (*p* = 0.589).

**Conclusions:**

For patients who have distant lymph node or liver metastasis and those who will not undergo postoperative chemotherapy, non-curative gastric resection has no survival benefit.

## Background

The prognosis of stage IV gastric cancer is poor, with an expected survival period of 3–5 months without treatment [1]. The treatment strategies for patients with stage IV gastric cancer mainly consist of chemotherapy, palliative surgery, and symptomatic treatment. Although systemic chemotherapy has been reported to extend the overall survival (OS) of patients with stage IV gastric cancer [2], the impact of non-curative gastric resection as palliative surgery is still unknown. Meanwhile, despite improvement in preoperative examinations, the diagnosis of stage IV gastric cancer is occasionally made only after laparoscopic or open exploration. In these cases, surgeons have to make an intraoperative decision as to whether or not they perform a non-curative gastric resection. In this study, we investigated the impact and optimal indication of non-curative gastric resection for patients with stage IV gastric cancer that was diagnosed during surgery.

## Methods

### Patients

We examined all 1086 patients who underwent open abdominal surgery for gastric cancer between July 2004 and June 2014 at Asahi General Hospital, Chiba, Japan. Among these patients, 72 (6.6 %) were diagnosed with stage IV gastric cancer after open exploration that was preoperatively intended to be a radical excision procedure (R0 surgery). The objective of this study was to examine these 72 patients. Cases of emergency surgery for bleeding or perforation were excluded from this study.

### Strategies for stage IV gastric cancer diagnosed during the operation

When stage IV gastric cancer was diagnosed during the operation, in principle, we removed the primary lesion (total gastrectomy or gastric resection) as a volume reduction measure. When the tumor showed strong invasion to other organs such as the pancreas, esophagus, or duodenum, we did not perform the gastric resection, and a gastrojejunostomy was performed as necessary for the patients with a preoperative gastric obstruction. In principle, we initiated chemotherapy as soon as possible after surgery regardless of whether gastric resection was performed. Chemotherapy was not administered to patients who refused or had general health issues that precluded chemotherapy.

### Methods

These data were retrospectively analyzed based on patients’ medical records. The histological and pathological findings were described according to the International Union against Cancer (UICC) 7th edition of the tumor-node-metastasis (TNM) classification. The 72 patients were classified into two groups: patients who underwent a non-curative gastric resection (gastric resection group) and those who did not (no-resection group). We compared the baseline characteristics between the two groups. This study received a priori approval from the institutional review committee.

### Statistical analysis

The data are expressed as the median (range). Univariate and multivariate analyses for survival were performed. We analyzed the OS of all patients, and the subgroup analyses examined the optimal indications for non-curative gastric resection. The differences in surgical outcomes between the resection and non-resection groups were evaluated using Student’s *t* tests and chi-squared tests. A Cox proportional hazard model based on the uni- and multivariate analyses was used to assess the independent factors affecting OS. The survival analysis was calculated using a log-rank test. All statistical analyses were performed using the StatView software package (SAS Institute, Cary, NC), and differences with *p* < 0.05 were considered significant.

## Results

The baseline characteristics of all 72 patients are shown in Table 1. The median age was 68 years (range 22–87), and this study included 50 male and 22 female patients. The median follow-up period was 1.0 year (0.0–5.0 years). The non-curative factors that resulted in the diagnosis of stage IV cancer were peritoneal metastasis (P+) in 38 patients, positive peritoneal cytology (CY+) in 38, distant lymph node metastasis (LN+) in 11, and liver metastasis (H+) in 4. Postoperative chemotherapy was administered to 56 patients (77.8 %). A TS-1® (combination capsules of tegafur, gimeracil, and oteracil potassium, Taiho Pharmaceutical Co. Ltd, Japan) based regimen was the most commonly used first line chemotherapy (TS-1: *n* = 32 [57.1 %], TS1 + cisplatin: *n* = 12 [21.4 %]), followed by irinotecan + CDDP in 5 patients (8.9 %), paclitaxel in 4 patients (7.1 %), and other forms in 3 patients.

**Table 1:** Comparison of demographic and other characteristics between the gastric resection and no-resection groups. There were statistically significant differences in operation time, estimated amount of bleeding, initial oral intake, and complication rate

Characteristics	All (*n* = 72)	Gastric resection group (*n* = 44)	No-resection group (*n* = 28)	*p* value
Age	68 (22–87)	68 (32–87)	68 (22–87)	0.218
Gender (male to female ratio)	50:22	28:16	22:6	0.180
PS ≥ 2	8 (11.1 %)	5 (11.4 %)	3 (10.8 %)	0.931
ASA-PS ≥2	43 (59.7 %)	26 (59.1 %)	17 (60.8 %)	0.891
Residual stomach cancer	3 (41.7 %)	2/44 (4.5 %)	1/28 (3.6 %)	0.840
Incurable factor (P:CY:LN:H (include overlap))	38:38:11:4	21:24:7:2	17:14:4:2	–
Histological classifications(por:sig:tub:others)	36:12:21:3	20:6:16:2	16:6:5:1	0.371
Resection of other organs	Liver;4, T/C;3 sp;1, GB;7	Liver;4, T/C;2, spleen;1, GB;6	T/C 2; GB;1	–
Operation time (min)	225 (49–566)	254 (164–498)	115 (49–566)	<0.01
Estimated amount of bleeding (ml)	120 (0–1464)	266 (0–1457)	20 (0–1464)	<0.01
Initial oral intake (POD)	5 (1–8)	6 (2–7), impossible 1	4 (1–8)	<0.01
Complications rate (Clavian-Dindo ≥ II)	14 (19.4 %)	12 (27.3 %) (II:5, IIIa:6, V:1)	2 (7.1 %) (II:2)	0.036
Postoperative chemotherapy	56 (77.8 %)	35 (79.5 %)	21 (75.0 %)	0.651
Obstruction symptoms	4 (5.6 %)	2 (4.5 %)	2 (7.1 %)	0.639

*PS* performance status, *ASA*-*PS* ASA physical status, *por* poorly differentiated adenocarcinoma, *sig* signet cell adenocarcinoma, *tub* tubular adenocarcinoma, *POD* postoperative days, *T*/*C* transverse colon, *GB* gallbladder

Among the 72 patients who were diagnosed with stage IV gastric cancer during the operation, 44 received a non-curative gastric resection as a palliative surgery (14 gastrectomy and 30 total gastrostomy), 14 received a gastrojejunostomy for bypass surgery, and 14 patients received only a laparotomy. The reason precluding the gastric resection was invasion to the pancreas in 19 patients, to the esophagus in 4 patients, to the duodenum in 2 patients, and other reasons in 3 patients. Table 1 presents the comparison of the baseline characteristics between the gastric resection (*n* = 44) and no-resection groups (*n* = 28). There were no statistically significant differences in patient’s age, ASA-physical status, and performance status between the groups. In the gastric resection group, the operation time was longer and the estimated amount of bleeding was much higher than in the non-resection group (both *p* < 0.01). The complication rate (≥Clavian-Dindo class II) was higher in the gastric resection group (*p =* 0.036), and one patient died of anastomotic leakage of the transverse colon 16 days postoperatively. The percentage of patients who received chemotherapy was 79.5 % in the gastric resection group and 75.0 % in the non-resection group, and this was not significantly different (*p* = 0.651).

**Fig. 1:**
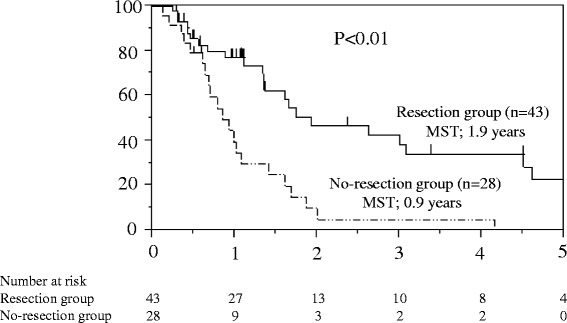
In the survival analysis (log-rank test), the median survival time (MST) in the resection group and no-resection groups were 1.9 and 0.9 years, respectively (*p* = 0.014). Overall survival (log-rank test)

In the survival analysis, the median survival time (MST) in the resection and non-resection groups were 1.9 and 0.9 years, respectively (*p* = 0.014) (Fig. 1). We utilized a Cox proportional hazard model based on the uni- and multivariate analyses to determine the independent factors affecting OS (Table 2). In the univariate analysis, we found that tubular adenocarcinoma, gastric resection, and postoperative chemotherapy were independent factors affecting the OS. We entered these three factors into the multivariate analysis, and found that volume reduction surgery (HR = 0.309; 95 % CI = 0.152–0.615) and postoperative chemotherapy (HR = 0.136; 95 % CI = 0.056–0.353) were influential.

**Table 2:** Cox proportional hazard model based on the uni- and multivariate analyses to determine the independent factors for the overall survival (OS). For the univariate analysis, we selected tubular adenocarcinoma, gastric resection, and postoperative chemotherapy as independent factors affecting the OS. Similarly, for the multivariate analysis, we selected volume reduction and postoperative chemotherapy as independent factors associated with OS

Univariate analysis	Hazard ratio	95 % CI	*p* value
Male	0.976	0.518–1.944	0.941
Age ≥ 68	1.070	0.584–1.954	0.825
PS ≥ 2	2.329	0.990–4.881	0.053
ASA-PS ≥ 2	1.156	0.623–2.179	0.648
Tubular adenocarcinoma	0.483	0.231–0.935	0.030
P(+) and/or CY (+)	0.886	0.448–1.913	0.744
LN(+) or H(+)	0.970	0.476–1.851	0.928
Non-curative gastric resection	0.326	0.172–0.614	<0.01
Complications (Clavian-Dindo ≥ 2)	1.820	0.832–3.679	0.127
Postoperative chemotherapy	0.260	0.120–0.626	0.004
Multivariate analysis	Hazard ratio	95 % CI	*p* value
Tubular adenocarcinoma	0.531	0.233–1.130	0.102
Non-curative gastric resection	0.309	0.152–0.615	<0.01
Postoperative chemotherapy	0.136	0.056–0.353	<0.01

*PS* performance status, *ASA*-*PS* ASA physical status

In the subgroup analysis of OS in patients who had P(+) and/or CY(+) with LN(−)H(−), the resection group had a longer survival time, whereas no survival benefit was obtained from the non-curative resection in LN(+) or H(+) patients (Table 3). In the subgroup analyses based on histological classification, in patients with tubular adenocarcinoma, the resection group showed a longer survival time. However, patients with other types of gastric cancer, including poorly differentiated adenocarcinoma and signet cell adenocarcinoma, did not experience a survival benefit from the non-curative resection. For patients who received chemotherapy after surgery, the resection group showed a longer survival time, whereas no survival benefit was obtained for patients who did not receive postoperative chemotherapy.

**Table 3:** Summary of the results of the survival analysis (log-rank test). Overall, the gastric resection group showed a longer survival time. However, gastric resection provided no survival benefit for patients who have distant lymph node or liver metastasis, non-tubular adenocarcinoma, or did not undergo postoperative chemotherapy

Condition	Gastric resection group MST (years)	No-resection group MST (years)	*p* value (log-rank test)
• Entirely	1.9 (*n* = 43)	0.9 (*n* = 28)	<0.01
• Incurable factors			
P(+) and/or CY (+), LN(−)H(−)	2.6 (*n* = 34)	0.8 (*n* = 21)	<0.01
P(−)CY(−), LN (+) or H (+)	1.7 (*n* = 7)	1.6 (*n* = 5)	0.527
• Histological classifications			
Tubular adenocarcinoma	1.9 (*n* = 16)	0.6 (*n* = 10)	0.042
Poorly differentiated adenocarcinoma	2.6 (*n* = 28)	1.0 (*n* = 18)	0.318
Signet cell adenocarcinoma	1.4 (*n* = 5)	0.8 (*n* = 6)	0.070
• Postoperative chemotherapy			
Chemotherapy (+)	3.0 (*n* = 36)	0.9 (*n* = 21)	<0.01
Chemotherapy (−)	0.7 (*n* = 8)	0.2 (*n* = 7)	0.589

We combined the subgroup analyses to investigate the interaction of LN(+) or H(+) and with non-tubular adenocarcinoma (Table 4). The patients who had CY(+) and/or P(+) with LN(−) P(−) showed a survival benefit despite the histological type, while patients who had LN(+) or H(+) showed no survival benefit independent of the histological type.

**Table 4:** Subgroup analysis of the overall survival. Despite the histological type, a survival benefit from gastric resection was observed in patients who did not have distant lymph node or liver metastasis

Condition	Tubular adenocarcinoma		Non-tubular adenocarcinoma (por, sig, others)	
P(+) and/or CY(+), LN(−)H(−)	Resection (*n* = 9) MST; 3.1 years	No-resection (*n* = 3) MST; 0.6 years	Resection (*n* = 25) MST; 1.4 years	No-resection (*n* = 18) MST; 0.8 years
	*p* < 0.01		*p* = 0.034	
P(−)CY(−), LN(+) or H(+)	Resection (*n* = 5) MST; 1.7 years	No-resection (*n* = 2) MST; 1.2 years	Resection (*n* = 1) survival time; 1.0 years	No-resection (*n* = 3) MST; 1.6 years
	*p* = 0.819		–	

We performed Fisher’s exact test to examine the relationship between the presence of LN(+) or H(+) and the rate of postoperative chemotherapy. In patients with LN(+) or H(+), postoperative chemotherapy was performed in 14 and not performed in 2. Meanwhile, postoperative chemotherapy was performed in 42 patients and not performed in 13 patients who have LN(−)H(−). Postoperative chemotherapy was performed regardless of the existence of LN(+) or H(+) (*p* = 0.501).

## Discussion

Gastric cancer is the second leading cause of cancer-related deaths in Japan [3]. Complete R0 resection and negative lymph nodes are the most important factors for long-term survival. However, various factors render gastric cancer incurable in many patients. Radical excision (R0 surgery) is impossible in stage IV advanced gastric cancer, and the treatment of the patient mainly consists of chemotherapy, palliative surgery, and symptomatic treatment [4–6]. The prognosis of advanced gastric cancer remains poor despite improvements in its treatment over the last two decades [7], and the expected survival period of untreated stage IV gastric cancer was reported to be 3–5 months [1]. Systemic chemotherapy alone has been reported to extend the OS by up to 9–11 months [2]. In addition, the molecular targeting agent trastuzumab has also been reported to prolong OS by 11–14 months; however, the survival benefit of chemotherapy alone is limited.

The efficacy and indication of palliative surgery for incurable gastric cancer remain controversial [8–10]. Although some studies have demonstrated a survival benefit of non-curative gastrectomy [11, 12], most retrospective analyses show no survival benefit and recommend that palliative gastrectomy be performed if patients have tumor-related symptoms [13–16]. Although retrospective studies by nature have inherent selection bias and some confusion exists regarding their interpretation, the indication of non-curative gastric resection for stage IV gastric cancer is currently strictly restricted [17].

Two randomized, controlled trials have been conducted that may provide further insight into the survival benefits of non-curative gastrectomy. Rudloff and colleagues reported the results of the GYMSSA trial and concluded that complete cytoreductive surgery combined with hyperthermic intraperitoneal chemotherapy and systemic chemotherapy may improve OS more than systemic chemotherapy alone [18]. A prospective randomized trial (REGATTA; JCOG0705/KGCA01) was initiated in three Asian countries in 2008 and was designed to compare gastrectomy plus chemotherapy to chemotherapy alone in advanced gastric cancer with a single non-curative factor [19]. Currently, no large-scale randomized controlled trial has denied the benefit of non-curative gastric resection for stage IV gastric cancer, making it important to select patients who may experience a survival benefit of this procedure.

Tokunaga and coworkers did not find a survival benefit of palliative gastrectomy in patients with peritoneal metastasis [16]. Chen et al. reported that palliative gastrectomy combined with hepatectomy improved the OS of patients who had stage IV gastric cancer with liver metastases [13]. Our study demonstrated that the independent factors related to OS were non-curative gastric resection and postoperative chemotherapy. Although the OS of the gastric resection group was significantly longer than the no-resection group, this result was not surprising because cases in the no-resection group had more severe invasion to the other organs. In this study, gastric resection was performed when invasion to the other organs was minimal and the procedure was possible. Therefore, the resection group had the potential for longer survival than the no-resection group, and our result may reflect the impact of invasion to other organs, i.e., the existence of resectability, rather than the effect of the procedure itself. In subgroup analyses, although we found longer survival times in patients whose incurable factors were P(+) and/or CY(+), it remains unknown how the resection itself influenced the survival time. Meanwhile, we found no prognostic difference between the gastric resection and no-resection groups in cases whose incurable factors were LN(+) or H(+) and in patients who did not receive postoperative chemotherapy. It is worth noting that patients with LN(+) or H(+) and those who did not undergo postoperative chemotherapy received no survival benefit from non-curative gastric resection even though the OS of the gastric resection group was significantly longer. Although we suspected that non-tubular adenocarcinoma was also the independent risk factor that opposes the surgical resection, we considered that LN(+) or H(+) more strongly influenced to OS.

This study has some limitations. As in all retrospective investigations, selection bias is present in this study. The different surgeons judged whether non-curative gastric resection was possible or not. To clarify the veridical efficacy of non-curative gastric resection, we should compare patients with and without gastric resection among patients in whom gastric resection is possible. Another limitation is the non-standardized nature of the chemotherapy regimen, which differed over time and sometimes by case. In addition, we could not evaluate the impact of HER-2 positive or not, although it affects the prognosis, and involved also in the selection of treatment [4, 5]. Quality of life is one of the most important endpoints for individuals with advanced gastric cancer [20]; however, we could not measure it prospectively. Further studies involving more standardized and prospective analyses are needed.

## Conclusions

In conclusion, we believe that this study provides one indicator to determine whether non-curative gastric resection should be performed when stage IV gastric cancer is diagnosed during the operation. Non-curative gastric resection for stage IV gastric cancer may provide a survival benefit for patients whose incurable factors are P(+) or CY(+) and postoperative chemotherapy is performed. Meanwhile, there was no survival benefit for patients who have LN(+) or H(+) and who did not undergo postoperative chemotherapy; therefore, it should be strictly restricted in these circumstances.

### Ethics

This study was approved by the ethical committee of Asahi General Hospital and the reference number was 2014091620. Informed consent and consent to publish were obtained from the patients or their families.

## Abbreviations

CI: confidence interval
CY(+): positive peritoneal cytology
H(+): liver metastasis
HR: hazard ratio
LN(+): distant lymph node metastasis
MST: median survival time
OS: overall survival
P(+): peritoneal metastasis
